# Comparative Detection and Inter-Modality Agreement of Pulp Stones Using Digital Periapical Radiography and CBCT at Two Voxel Sizes: An Ex Vivo Study

**DOI:** 10.3390/diagnostics16070961

**Published:** 2026-03-24

**Authors:** Hassan Hamed Kaabi, Sarah Saeed Binhassan, Sultan Hamad Alrumaih, Mohammed Jamal Alotaibi, Abdullah Khalid Bakarman, Nawaf Abdulaziz Alghamdi, Hamad Abdullah Almuhaythif, Qamar Mohammadziad Hashem, Abdulfatah Samih Alazmah

**Affiliations:** 1 Department of Oral Medicine and Diagnostic Sciences, College of Dentistry, King Saud University, P.O. Box 60169, Riyadh 11545, Saudi Arabia; 2 Department of Clinical Laboratory Sciences, College of Applied Medical Sciences, King Saud University, P.O. Box 10219, Riyadh 11433, Saudi Arabia; sbinhassan@ksu.edu.sa; 3 College of Dentistry, King Saud University, P.O. Box 60169, Riyadh 11545, Saudi Arabia; sultan_alrumaih@hotmail.com (S.H.A.); mjml1996m@gmail.com (M.J.A.); 441100754@student.ksu.edu.sa (A.K.B.); n3ghamdi@gmail.com (N.A.A.); 442100354@student.ksu.edu.sa (H.A.A.); 4 Department of Conservative Dental Sciences, College of Dentistry, Prince Sattam bin Abdulaziz University, Al-Kharj 11942, Saudi Arabia; qhashem@psau.edu.sa; 5 Department of Pediatric Dentistry, College of Dentistry, Prince Sattam bin Abdulaziz University, Al-Kharj 11942, Saudi Arabia

**Keywords:** cone beam computed tomography, pulp stone, pulp calcification, digital periapical radiography, voxel size, extracted teeth

## Abstract

**Background/Objectives:** Pulp stones are calcified masses within the dental pulp that may complicate endodontic procedures. Although cone beam computed tomography (CBCT) has been reported to detect pulp stones more frequently than two-dimensional radiography, direct comparisons between digital periapical radiography (DPR) and CBCT, particularly at different voxel sizes, remain limited. This study aimed to compare pulp stone detection rates between DPR and CBCT acquired at two voxel sizes and to evaluate inter-modality agreement using a location-based analysis for pulp stone identification in extracted teeth. **Methods:** Fifty-two extracted human teeth were examined using DPR and CBCT at voxel sizes of 0.2 mm and 0.1 mm under standardized ex vivo conditions. Pulp stones were evaluated in the coronal and radicular regions using a binary scoring system (presence/absence). Detection rates were compared across imaging modalities, and inter-modality agreement was evaluated using McNemar’s test in the analysis stratified by pulp stone location. Associations between pulp stone detection and age, gender, tooth status, and stone location were explored using chi-square tests. **Results:** CBCT at 0.1 mm demonstrated the highest detection rate for pulp stones (63.5%), followed by CBCT at 0.2 mm (57.7%) and DPR (50%), with no statistically significant differences among modalities (*p* > 0.05). Agreement analysis showed that CBCT at 0.2 mm had higher agreement with CBCT at 0.1 mm than DPR, particularly in the coronal region, whereas DPR showed lower agreement in the radicular region. No significant associations were observed between pulp stone detection and age, gender, or tooth status. **Conclusions:** Under standardized ex vivo conditions, CBCT showed numerically higher pulp stone detection rates than DPR. Location-based agreement analysis indicated greater consistency between CBCT voxel sizes than between CBCT and DPR, particularly in the radicular region. These findings highlight differences in pulp stone detectability across imaging modalities and voxel resolutions and may help explain variability in radiographic detection under controlled conditions.

## 1. Introduction

A pulp stone, or denticle, is a discrete calcified mass that can occur in the pulp cavity of any primary or permanent tooth. Pulp stones can occur in healthy, infected, and impacted [1] or unerupted teeth [2,3]. They are found more frequently in the pulp chamber, although they may also appear in the radicular pulp [4]. The exact cause of dental pulp calcification remains uncertain; however, factors such as aging [5], pulp degeneration [4], orthodontic tooth movement [6], and dental caries [7] are thought to contribute to its development.

Pulp stones can be classified based on their number (single or multiple), location (free, attached, or dentin-embedded), and structure (true or false). Histologically, true pulp stones resemble dentin, containing dentinal tubules surrounded by predentin and odontoblasts, whereas false pulp stones lack tubules and are composed of mineralized degenerating cells [8]. The mineralization pattern in the dental pulp varies by region, with the higher cellular content of the coronal pulp leading to nodular calcifications, whereas the more fibrous nature of the radicular pulp contributes to diffuse mineralization [9].

Dental pulp stones are clinically significant, particularly in endodontic therapy, where they can obstruct access during root canal treatment [10,11] and hinder effective disinfection [12]. Despite their presence, pulpal calcifications alone do not necessitate endodontic intervention unless accompanied by other signs or symptoms [8]. They have also been suggested as potential indicators of systemic conditions, including calcified atherosclerotic plaques [13], ischemic heart disease [14], type II diabetes and hypertension [15].

The reported prevalence of pulp stones varies widely, ranging from 8% to 98%, with these differences attributed to ethnic and geographical factors as well as variations in detection methods [12,16,17,18,19]. Traditional two-dimensional imaging techniques, such as periapical, bitewing, and panoramic radiographs, are limited to detecting pulp stones larger than 200 μm and therefore often underestimate their true prevalence [17]. Histological surveys, although they show higher prevalence rates, are invasive because they require tooth extraction and are limited to analyzing small sections, which may also lead to underestimation [8,20,21].

Given these limitations, three-dimensional cone beam computed tomography (CBCT) provides enhanced visualization of pulp stones compared with conventional two-dimensional radiographs [22,23] and represents a less invasive alternative to histological examination [18,22]. In recent years, it has been increasingly used to support complex dental procedures and improve diagnostic accuracy [24]. In endodontics, CBCT is particularly valuable for managing complex cases by enabling detailed examination of root canal systems and precise localization of calcified canals [24]. CBCT units provide voxel sizes ranging from 0.075 to 0.4 mm, with voxel size selection influencing scanning time, reconstruction duration, diagnostic accuracy, and radiation dose [25,26]. Smaller voxel sizes are associated with improved visualization of finer details and smaller defects, thereby enhancing diagnostic precision [27].

Previous studies have investigated the prevalence of pulp stones using various imaging modalities [16,18,20,24], with some comparing two-dimensional panoramic radiographs with CBCT for their detection [22,23]. However, direct comparisons between digital periapical radiography (DPR) and CBCT under standardized conditions remain limited. Furthermore, the influence of CBCT voxel size on pulp stone detectability and inter-modality agreement remains insufficiently investigated.

Accurate identification of pulp stones is clinically essential, as undetected calcifications may complicate endodontic access, increase procedural difficulty, and adversely affect treatment outcomes [10,11]. In addition, their potential association with systemic conditions further emphasizes their broader clinical relevance [2,13,14,15,16,28].

Therefore, this study aimed to compare pulp stone detection rates between DPR and CBCT acquired at two voxel sizes (0.1 mm and 0.2 mm) in extracted human teeth. We hypothesized that CBCT, particularly at smaller voxel sizes, would demonstrate higher detection rates than DPR. Secondary analyses examined differences according to gender, age, tooth status (carious vs. non-carious), and location (coronal vs. radicular), followed by evaluation of inter-modality agreement stratified by location.

## 2. Methods

### 2.1. Study Design and Ethical Approval

This prospective observational ex vivo study assessed the detection of dental pulp stones using DPR and CBCT at two voxel sizes (0.1 mm and 0.2 mm) in extracted human teeth. This study involving extracted human teeth was approved by the Institutional Review Board (IRB) of the Research Ethics Committee at King Saud University, Riyadh, Saudi Arabia (IRB No. E-23-8063), and registered with the College of Dentistry Research Center (CDRC No. IR 0479). Written informed consent was obtained from all participants for the donation of their extracted teeth for research purposes. The purpose of the study and procedures involved were clearly explained to each participant. The study was conducted in accordance with the ethical principles outlined in the Declaration of Helsinki (1964), as revised in 2013.

### 2.2. Sample Selection

Extracted permanent teeth were obtained from Saudi patients aged 18 years or older who attended the Oral Surgery Clinic at King Saud University Dental Hospital, Riyadh, Saudi Arabia, and required tooth extraction for clinical reasons. Teeth were consecutively collected during the study period and included anterior, premolar, and molar teeth from both the maxillary and mandibular arches to ensure representation of different tooth types. Extractions were performed for various clinical indications, including advanced caries, periodontal disease, or orthodontic purposes.

The extracted teeth were brushed under running water to remove blood and debris, followed by the removal of any attached bone or soft tissue. The samples were then preserved in 10% neutral buffered formalin until imaging. Teeth were included regardless of pulp stone status and selected based on structural integrity and suitability for radiographic assessment.

Teeth with previous root canal treatment, prosthetic crowns, fractures involving the pulp, or incomplete or resorbed roots were excluded. After applying these criteria, 52 teeth were eligible for analysis. No formal sample size calculation was performed, as the study was based on the availability of extracted teeth meeting the inclusion criteria during the study period. The presence or absence of pulp stones was determined only after imaging using DPR and CBCT at two voxel sizes. The final sample comprised 42.3% maxillary teeth (6 anterior, 16 posterior) and 57.7% mandibular teeth (6 anterior, 24 posterior).

### 2.3. Imaging Procedures

All imaging procedures were performed by a qualified and licensed X-ray technician under standardized conditions. Each extracted tooth was embedded in a wax block to ensure stable positioning and reproducible alignment during both DPR and CBCT imaging. Teeth were consistently oriented with their long axis perpendicular to the image receptor for DPR and centered within the field of view for CBCT to maintain uniform geometry across imaging modalities.

Identical positioning and handling protocols were applied for all imaging procedures to ensure comparability between modalities. No bone or soft-tissue simulation was used in order to minimize variability and allow direct comparison of pulp stone detectability between imaging methods under controlled ex vivo conditions.

### 2.4. Digital Periapical Radiography (DPR)

DPR radiographs were acquired using the ProX™ digital radiography system (Planmeca, Helsinki, Finland) under standardized exposure parameters (6 mA, 63 kV). Images were obtained following the manufacturer’s recommended exposure settings for periapical imaging. Radiographs were displayed on a 34-inch LED monitor in a dimly lit room and evaluated using Romexis software (version 5.2.R; Planmeca, Helsinki, Finland) at a resolution of 2560 × 1600 pixels. Image brightness, contrast, and optical density were adjusted using the software’s built-in tools to optimize visualization during assessment.

### 2.5. Cone Beam Computed Tomography (CBCT)

CBCT imaging was performed using a ProMax 3D Max unit (Planmeca, Helsinki, Finland). Each extracted tooth was scanned at two voxel sizes (0.1 mm and 0.2 mm) under identical acquisition conditions to allow direct comparison of pulp stone detectability.

Both voxel size protocols were acquired using a tube voltage of 82 kV and a tube current of 6 mA. The exposure times were 12.27 s for the 0.1 mm voxel size and 12.14 s for the 0.2 mm voxel size. A consistent field of view (5.0 cm × 5.7 cm) was used for both scans.

All images were reconstructed using the default manufacturer settings, which were kept consistent across datasets. This ensured that differences in pulp stone detectability were primarily related to voxel size rather than variability in imaging conditions.

The 0.1 mm voxel size generated 571 image slices, whereas the 0.2 mm voxel size generated 286 slices, reflecting the higher spatial resolution of the smaller voxel size. CBCT datasets were evaluated in axial, sagittal, and coronal planes using the same display conditions and software (Romexis, Planmeca, Helsinki, Finland) used for DPR image analysis.

### 2.6. Imaging Analysis

**Figure 1:**
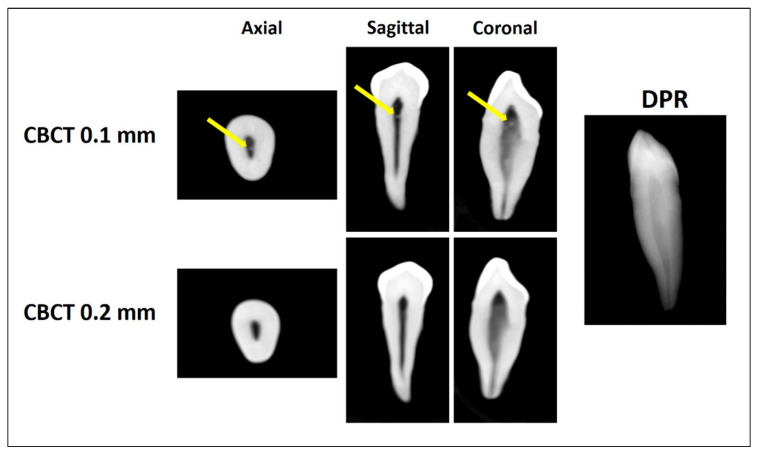
Pulp stone detection using CBCT 0.1 mm, CBCT 0.2 mm (axial, sagittal, and coronal planes), and DPR. The arrows indicate a coronal pulp stone detected by CBCT 0.1 mm but not by CBCT 0.2 mm or DPR.

**Figure 2:**
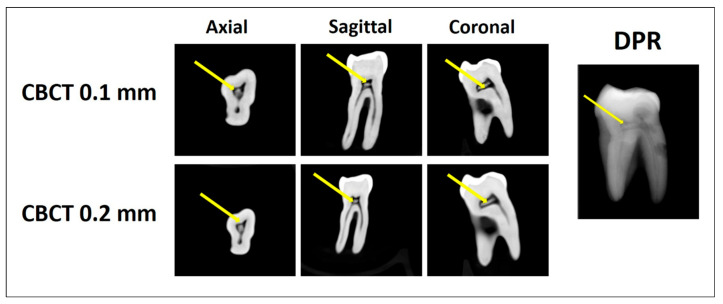
Pulp stone detection using CBCT 0.1 mm, CBCT 0.2 mm (axial, sagittal, and coronal planes), and DPR. The arrows indicate a coronal pulp stone detected by all methods.

**Figure 3:**
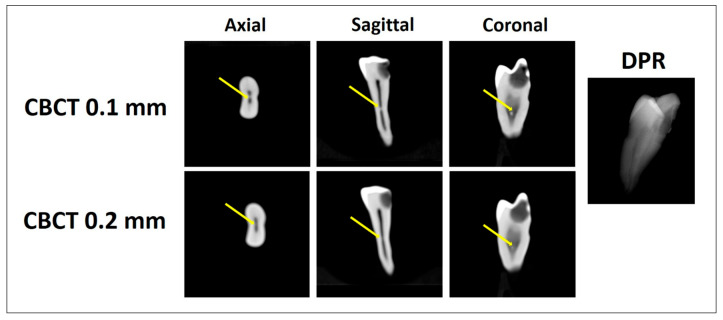
Pulp stone detection using CBCT 0.1 mm, CBCT 0.2 mm (axial, sagittal, and coronal planes), and DPR. The arrows indicate a radicular pulp stone detected by CBCT 0.1 mm and CBCT 0.2 mm but not by DPR.

Two trained investigators independently evaluated each tooth for the presence or absence of pulp stones using all three imaging modalities (DPR, CBCT at 0.2 mm voxel size, and CBCT at 0.1 mm voxel size). For each tooth, the pulp space was divided into coronal and radicular regions using the cementoenamel junction as the anatomical reference. Pulp stones were defined as discrete radiopaque structures within the pulp space (Figure 1, Figure 2 and Figure 3). Pulp stones were evaluated using a binary scoring system (presence/absence), consistent with methodologies used in previous pulp stone imaging studies (5, 30, 31). Each imaging modality was assessed independently, and the order of image evaluation was randomized for each examiner to minimize potential learning or recall bias. Inter-examiner agreement was assessed using Cohen’s kappa statistics (κ = 0.80), while intra-examiner reliability demonstrated high consistency (κ = 0.80 for examiner 1 and κ = 0.78 for examiner 2). In cases of disagreement, a consensus was achieved through joint image review.

### 2.7. Statistical Analysis

Statistical analysis was performed using the Statistical Package for the Social Sciences (SPSS) software (version 25, IBM Corp., Armonk, NY, USA). Descriptive statistics, including frequencies and percentages, were used to summarize pulp stone detection across imaging modalities. Differences in detection rates according to gender, age group, tooth status, and pulp stone location were assessed using chi-square tests.

Inter-modality agreement between imaging methods was evaluated using McNemar’s test, with analyses stratified by pulp stone location (coronal vs. radicular). In this location-based analysis, CBCT at a voxel size of 0.1 mm was used as a comparative reference due to its higher spatial resolution, rather than as a gold standard. Inter- and intra-examiner reliability were assessed using Cohen’s kappa statistics. A significance level of *p* < 0.05 was applied for all statistical tests.

## 3. Results

The study sample comprised 52 extracted permanent teeth obtained from patients across different age groups, genders, and tooth conditions. Teeth were extracted from patients aged 51–78 years (*n* = 22, 42.3%), followed by 36–50 years (*n* = 19, 36.5%) and 18–35 years (*n* = 11, 21.2%). With respect to gender, 34 teeth (65.4%) were obtained from male patients and 18 (34.6%) from female patients. Based on tooth status, 27 teeth (51.9%) were carious and 25 teeth (48.1%) were non-carious. The distribution of teeth by location included 6 maxillary anterior, 16 maxillary posterior, 6 mandibular anterior, and 24 mandibular posterior teeth.

DPR identified pulp stones in 50% (26/52) of teeth, CBCT at 0.2 mm voxel size in 57.7% (30/52), and CBCT at 0.1 mm voxel size in 63.5% (33/52). These differences were not statistically significant (*p* = 0.380), suggesting similar detection rates across imaging modalities. Although CBCT identified a higher number of teeth with pulp stones than DPR, these findings reflect differences in detection frequency rather than inter-modality agreement.

Pulp stone detection rates did not differ significantly between male and female teeth for any imaging modality (Table 1). Detection rates were comparable between genders across all modalities, with CBCT at 0.1 mm demonstrating the highest overall detection.

Pulp stone detection rates across age groups are presented in Table 2. Detection rates were generally higher in the middle-aged and older groups than in the youngest group across all imaging modalities; however, these differences were not statistically significant. A borderline increase with age was observed for DPR (*p* = 0.051), while no significant differences were found for CBCT at 0.2 mm (*p* = 0.436) or 0.1 mm (*p* = 0.826).

Pulp stone detection rates did not differ significantly between carious and non-carious teeth across imaging methods (Table 3). Although DPR showed higher detection in non-carious teeth, CBCT demonstrated higher detection in carious teeth; however, these differences were not statistically significant (*p* > 0.05) and should be interpreted with caution.

Pulp stone detection rates according to pulp location are presented in Table 4. Across all imaging modalities, pulp stones were detected more frequently in the coronal region than in the radicular region. Significant differences between locations were observed for DPR (*p* = 0.003) and CBCT at 0.2 mm (*p* = 0.041), whereas the difference was not statistically significant for CBCT at 0.1 mm (*p* = 0.076).

Inter-modality agreement by pulp stone location is summarized in Table 5. For coronal pulp stones, DPR showed moderate agreement with CBCT at 0.1 mm (64.3%; *p* = 0.454), whereas CBCT at 0.2 mm demonstrated higher agreement (85.7%; *p* = 0.125). In the radicular region, agreement between DPR and CBCT at 0.1 mm was lower (38.9%) and statistically significant (*p* = 0.022), whereas CBCT at 0.2 mm showed higher agreement (72.2%), which was not statistically significant (*p* = 0.063). These findings reflect relative agreement between imaging modalities rather than diagnostic accuracy, as no definitive gold standard was used.

## 4. Discussion

Dental pulp stones are common but clinically important findings, especially in endodontic practice. They may obstruct canal access, increase the risk of instrument separation, and compromise effective disinfection during root canal therapy [10,11,12]. While their presence alone does not justify intervention in the absence of other clinical signs [8], several studies suggest possible associations with systemic conditions such as calcified atherosclerotic plaques, ischemic heart disease, diabetes, and hypertension, as well as long-term medication use [2,13,14,15,16,28]. Accurate detection of pulp stones is important for treatment planning and anticipating technical challenges [10,11,12], and may also help identify patients with a tendency toward ectopic calcification and associated systemic conditions [2,13,28].

Imaging plays a key role in the visualization of pulp stones. Although numerous studies have investigated pulp stone prevalence using different imaging modalities, direct comparisons between DPR and CBCT remain limited. Furthermore, the influence of CBCT voxel size on pulp stone detectability has not been adequately explored. Therefore, the present ex vivo study compared pulp stone detection rates between DPR and CBCT acquired at two voxel sizes (0.1 mm and 0.2 mm) in extracted human teeth under standardized conditions.

Detection rates varied across imaging methods, with CBCT at 0.1 mm identifying the highest prevalence (63.5%), followed by CBCT at 0.2 mm (57.7%) and DPR (50.0%). These differences were not statistically significant and may reflect limited statistical power rather than true equivalence between modalities. Previous studies have reported a broad prevalence range (8–95%) [4,7,16,18,19,29], likely due to differences in imaging modality, voxel resolution, study design (in vivo vs. ex vivo), and tooth selection. CBCT-based studies, particularly those using smaller voxel sizes, consistently report higher detection rates than conventional two-dimensional radiographs [4,7,16,18,19,22,23,29], likely due to reduced anatomical superimposition and improved three-dimensional visualization. In agreement with these findings, CBCT at 0.2 mm demonstrated detection patterns more similar to CBCT at 0.1 mm than DPR, highlighting the advantage of three-dimensional imaging in pulp stone visualization.

CBCT systems offer a range of voxel sizes (approximately 0.075–0.4 mm), which influence scanning time, image quality, and radiation dose [25,26]. In this study, a smaller voxel size was associated with higher pulp stone detection, with CBCT at 0.1 mm showing the highest rate. This is consistent with previous reports that smaller voxels improve spatial resolution and detection of fine anatomical details [27]. However, this advantage must be balanced against increased radiation exposure and longer acquisition times in clinical practice [24].

Pulp stone detection rates did not differ significantly between genders across all imaging modalities. Previous studies have also reported no consistent gender association in pulp stone prevalence [22,29,30,31,32]. However, some studies report higher prevalence in females [33,34,35,36], potentially related to factors such as increased bruxism and hormonal influences, whereas others report higher prevalence in males [16,37], possibly due to greater pulpal inflammation [38]. Overall, these inconsistencies indicate that gender is not a reliable determinant of pulp stone formation and that observed differences likely reflect population or methodological variations.

Pulp stone detection showed a tendency to increase in the middle-aged and older groups across imaging methods; however, these differences were not statistically significant and should be interpreted cautiously. The highest detection rates were observed in the 36–50 age group. Reduced detectability in older individuals may be related to ongoing dentin deposition, which can incorporate pulp stones into the surrounding dentin and limit their radiographic visibility [8]. Previous studies report inconsistent associations between age and pulp stone formation, with some suggesting higher prevalence with increasing age due to cumulative pulpal changes or systemic factors [5,29,39,40], while others report no significant relationship [16,20,31,35,41].

Pulp stone detection did not differ significantly between carious and non-carious teeth across imaging modalities (*p* > 0.05). Although DPR showed higher detection in non-carious teeth, CBCT demonstrated higher detection in carious teeth, suggesting an influence of imaging modality on observed patterns. Previous studies report conflicting findings regarding this association, likely due to methodological and imaging differences [4,12,16,22,23,24,29,34,42,43].

Pulp stones were more frequently detected in the coronal than in the radicular region across all imaging modalities. Agreement analysis showed higher consistency between CBCT voxel sizes than between CBCT and DPR. In the radicular region, DPR demonstrated significantly lower agreement, mainly due to discrepancies in identifying the absence rather than the presence of pulp stones. These findings differ from previous reports of higher radicular detection on panoramic radiographs [23]. This discrepancy may be explained by methodological differences, including the inclusion of diffuse calcifications and anatomical variations that may mimic pulpal narrowing on two-dimensional images.

Pulp stones have important clinical implications. They may complicate root canal treatment by obstructing access, increasing procedural difficulty, and affecting disinfection, particularly in cases involving multiple calcifications [10,11,12]. They have also been associated with systemic conditions involving pathological calcification, including atherosclerosis, kidney stones, vascular calcifications, and osteoarthritis, suggesting a link with generalized mineralization processes. Osteopontin has been proposed as a mediator in pulp stone formation and is also involved in these conditions, supporting shared underlying pathways [13]. Although the distinction between single and multiple stones remains unclear, a greater number of affected teeth may indicate a systemic predisposition; however, the evidence remains observational [2,13,28].

In this study, CBCT acquired at a voxel size of 0.1 mm was used as a comparative reference in the location-based agreement analysis rather than a definitive gold standard. This choice reflects its higher spatial resolution and ability to provide three-dimensional visualization of intrapulpal calcifications. Although histology remains the traditional reference method, it is invasive and limited to selected tissue sections [44,45]. Micro-computed tomography (micro-CT) offers superior resolution but is limited by high cost, long acquisition times, and technical complexity [45,46]. Therefore, despite its lower resolution, CBCT represents a practical and clinically applicable reference for comparative imaging studies.

Although extracted teeth do not fully replicate in vivo conditions, they allow for standardized imaging and reduced variability in positioning and exposure. This controlled setting enabled direct comparison between DPR and CBCT by minimizing confounding factors such as anatomical superimposition. However, the absence of surrounding bone and soft tissues limits clinical simulation and may increase the apparent visibility of pulp stones, particularly on two-dimensional radiographs [47]. Consequently, the detection rates observed in this study may be higher than those encountered under routine clinical conditions. This effect is likely more pronounced for DPR due to anatomical superimposition and attenuation inherent to two-dimensional imaging [48]. In contrast, CBCT provides three-dimensional visualization with reduced superimposition, although its image quality and detectability remain influenced by voxel size and image noise [49,50]. Therefore, these findings should be interpreted with caution in clinical practice, where detection, particularly of small or radicular pulp stones, may be more challenging.

Several methodological limitations should be considered. The use of CBCT at 0.1 mm as a comparative reference, rather than a true gold standard such as histology or micro-CT, limits conclusions regarding diagnostic accuracy. The ex vivo design without surrounding bone or soft-tissue simulation represents an idealized imaging environment that may overestimate pulp stone detectability. In addition, the relatively small sample size and the lack of an a priori sample size calculation should be considered when interpreting the findings. However, the within-sample design, in which each tooth was evaluated across all imaging modalities, may help reduce inter-sample variability. Finally, the use of binary detection without assessment of stone size or observer confidence limited a more detailed evaluation.

## 5. Conclusions

Within the limitations of this ex vivo study, differences in pulp stone visualization were observed; however, the findings should be interpreted cautiously due to methodological constraints. Detection rates were highest with CBCT at 0.1 mm, followed by CBCT at 0.2 mm and DPR, although these differences were not statistically significant. In the location-based analysis, CBCT at 0.2 mm showed greater agreement with CBCT at 0.1 mm than DPR, particularly for radicular pulp stones. Overall, these findings suggest that selection of imaging modality and CBCT voxel resolution should be guided by clinical context and diagnostic needs while considering radiation exposure.

## Figures and Tables

**Table 1:** Distribution of pulp stone detection according to gender.

Method	Male (*n* = 34)		Female (*n* = 18)		*p*
	Pulp Stone				
	Yes	No	Yes	No	
DPR	17 (50%)	17 (50%)	9 (50%)	9 (50%)	1
CBCT 0.2 mm	20 (58.8%)	14 (41.2%)	10 (55.6%)	8 (44.4%)	0.820
CBCT 0.1 mm	22 (64.7%)	12 (35.3%)	11 (61.1%)	7 (38.9%)	0.798

Significance level < 0.05; *p*: *p* value (chi-square test).

**Table 2:** Distribution of pulp stone detection across age groups.

Method	18–35		36–50		51–78		*p*
	*n* = 11		*n* = 19		*n* = 22		
	Pulp Stone						
	No	Yes	No	Yes	No	Yes	
DPR	9 (81.8%)	2 (18.2%)	7 (36.8%)	12 (63.2%)	10 (45.5%)	12 (54.5%)	0.051
CBCT 0.2 mm	6 (54.5%)	5 (45.5%)	6 (31.6%)	13 (68.4%)	10 (45.5%)	12 (54.5%)	0.436
CBCT 0.1 mm	4 (36.4%)	7 (63.6%)	6 (31.6%)	13 (68.4%)	9 (40.9%)	13 (59.1%)	0.826

Significance level < 0.05; *p*: *p* value (chi-square test).

**Table 3:** Distribution of pulp stone detection according to tooth status.

Method	Non-Carious		Carious		*p*
	*n* = 25		*n* = 27		
	Pulp Stone				
	No	Yes	No	Yes	
DPR	10 (40%)	15 (60%)	16 (59.3%)	11 (40.7%)	0.165
CBCT 0.2 mm	12 (48%)	13 (52%)	10 (37%)	17 (63%)	0.424
CBCT 0.1 mm	10 (40%)	15 (60%)	9 (33.3%)	18 (66.7%)	0.618

Significance level < 0.05; *p*: *p* value (chi-square test).

**Table 4:** Distribution of pulp stone detection according to location within the dental pulp (coronal vs. radicular).

Method	Coronal		Radicular		*p*
	*n* = 52		*n* = 52		
	Pulp Stone				
	No	Yes	No	Yes	
DPR	28 (53.8%)	24 (46.2%)	43 (82.7%)	9 (17.3%)	0.003
CBCT 0.2 mm	28 (53.8%)	24 (46.2%)	39 (75%)	13 (25%)	0.041
CBCT 0.1 mm	24 (46.2%)	28 (53.8%)	34 (65.4%)	18 (34.6%)	0.076

Significance level < 0.05; *p*: *p* value (chi-square test).

**Table 5:** Inter-modality agreement between DPR and CBCT at 0.2 mm with CBCT at 0.1 mm for pulp stone detection, stratified by pulp stone location.

Stone Location		CBCT 0.1 mm—*n* (%)		Total	*p*
		No Stone	With Stone		
	DPR—*n* (%)				
Coronal	No stone	18 (75.0%)	10 (35.7%)	28 (53.8%)	0.454
	With stone	6 (25.0%)	18 (64.3%)	24 (46.2%)	
	Total	24 (100%)	28 (100%)	52 (100%)	
Radicular	No stone	32 (94.1%)	11 (61.1%)	43 (82.7%)	0.022
	With stone	2 (5.9%)	7 (38.9%)	9 (17.3%)	
	Total	34 (100%)	18 (100%)	52 (100%)	
	CBCT 0.2 mm—*n* (%)				
Coronal	No stone	24 (100%)	4 (14.3%)	28 (53.8%)	0.125
	With stone	0 (0%)	24 (85.7%)	24 (46.2%)	
	Total	24 (100%)	28 (100%)	52 (100%)	
Radicular	No stone	34 (100%)	5 (27.8%)	39 (75%)	0.063
	With stone	0 (0%)	13 (72.2%)	13 (25%)	
	Total	34 (100%)	18 (100%)	52 (100%)	

Significance level < 0.05; *p*: *p* value (McNemar’s test).

## Data Availability

The dataset supporting the findings of this study is available in Zenodo at: https://doi.org/10.5281/zenodo.19096294 (accessed on 1 March 2026).

## Abbreviations

CBCT	Cone beam computed tomography
DPR	Digital periapical radiography
IRB	Institutional Review Board
CDRC	College of Dentistry Research Center
SPSS	Statistical Package for the Social Sciences
IBM	International Business Machines Corporation
kV	Kilovolt
mA	Milliampere
mm	Millimeter
µm	Micrometer
cm	Centimeter
LED	Light-emitting diode
USA	United States of America
NY	New York
κ	Kappa
